# Identification and Validation of Key Genes of Differential Correlations in Gastric Cancer

**DOI:** 10.3389/fcell.2021.801687

**Published:** 2022-01-13

**Authors:** Tingna Chen, Qiuming He, Zhenxian Xiang, Rongzhang Dou, Bin Xiong

**Affiliations:** ^1^ Department of Gastrointestinal Surgery, Zhongnan Hospital of Wuhan University, Wuhan, China; ^2^ Hubei Key Laboratory of Tumor Biological Behaviors, Wuhan, China; ^3^ Hubei Cancer Clinical Study Center, Wuhan, China

**Keywords:** gastric cancer, differential correlation, switching mechanism, WGCNA, gene network

## Abstract

**Background:** Gastric cancer (GC) is aggressive cancer with a poor prognosis. Previously bulk transcriptome analysis was utilized to identify key genes correlated with the development, progression and prognosis of GC. However, due to the complexity of the genetic mutations, there is still an urgent need to recognize core genes in the regulatory network of GC.

**Methods:** Gene expression profiles (GSE66229) were retrieved from the GEO database. Weighted correlation network analysis (WGCNA) was employed to identify gene modules mostly correlated with GC carcinogenesis. R package ‘DiffCorr’ was applied to identify differentially correlated gene pairs in tumor and normal tissues. Cytoscape was adopted to construct and visualize the gene regulatory network.

**Results:** A total of 15 modules were detected in WGCNA analysis, among which three modules were significantly correlated with GC. Then genes in these modules were analyzed separately by “DiffCorr”. Multiple differentially correlated gene pairs were recognized and the network was visualized by the software Cytoscape. Moreover, GEMIN5 and PFDN2, which were rarely discussed in GC, were identified as key genes in the regulatory network and the differential expression was validated by real-time qPCR, WB and IHC in cell lines and GC patient tissues.

**Conclusions:** Our research has shed light on the carcinogenesis mechanism by revealing differentially correlated gene pairs during transition from normal to tumor. We believe the application of this network-based algorithm holds great potential in inferring relationships and detecting candidate biomarkers.

## Introduction

As an aggressive malignant tumor with poor prognosis and high mortality, gastric cancer (GC) is responsible for over 1,000,000 new cases and an estimated 768,000 deaths in 2020, making it the fifth in terms of incidence and fourth in terms of mortality worldwide (Sung et al., 2021). GC ranks second and third in incidence and mortality in China, respectively (Chen et al., 2016). Despite traditional treatments such as chemotherapy and surgery, GC leads to recurrences within 2 years after surgery and poor long-term survival due to its early metastasis via the lymphatic system, blood, and peritoneum (Wu et al., 2003; D'Angelica et al., 2004). Genetic mutations play a significant role in the carcinogenesis of GC aside from environmental factors (Karimi et al., 2014). Studies have shown that oncogenes and tumor-suppressor genes, including E-cadherin, p16, and p53, can be used as biomarkers for diagnosis, prediction of sensitivity to treatment, and prognosis of GC (de Mello et al., 2021). Although GC molecular pathogenesis has considerably evolved over the years, much remains to be unraveled (Tan and Yeoh, 2015). Thus, it is essential to detect new biomarkers with most regulatory alterations in gene network to elucidate GC etiology, thereby providing information for a targeted treatment.

At this time, various bioinformatic methods have been developed based on gene expression data, which provide effective tools for the comprehensive analysis of the gene network in the pathogenesis of cancers (Zhang et al., 2018). In recent years, a variety of papers have reported and studied new biomarkers which possess a vital position in the gene network in different cancers such as, hepatocellular carcinoma (Fang et al., 2021), breast cancer (Yang et al., 2021), lung cancer (Chen et al., 2021) and bladder cancer (Liao et al., 2021). Usually, novel biomarkers were identified based on differentially expressed genes analysis (DEA) between disease and healthy tissues or status (Marco-Puche et al., 2019). However, what should be of concern is that the incidence of a disease is a combined effect of multiple highly interactive genes. Correlation analysis is an important approach for omics data and offers clues for gene regulatory networks (Eisen et al., 1998). As complementary to traditional analytical methods of gene expression data, it is essential to look at the alternation of gene correlation, referred to as “differential correlations”, in the pathogenesis of cancers (de la Fuente, 2010).

For the first time, we constructed an *in-silico* network in GC pathogenesis and identified two novel key genes based on the theory of ‘differential correlation’. Firstly, we detected 15 co-expressed genes modules by weighted gene co-expression network analysis (WGCNA). Then, the differential correlations of genes in the modules which were mostly correlated with GC were calculated. Furthermore, a gene network was built and functional analysis of key genes was carried out. Finally, the expression patterns of GEMIN5 and PFDN2, the two novel biomarkers in GC, were confirmed in the laboratory by RT-qPCR, WB, and IHC.

## Materials and Methods

### Data Collection and Preprocessing

**FIGURE 1:**
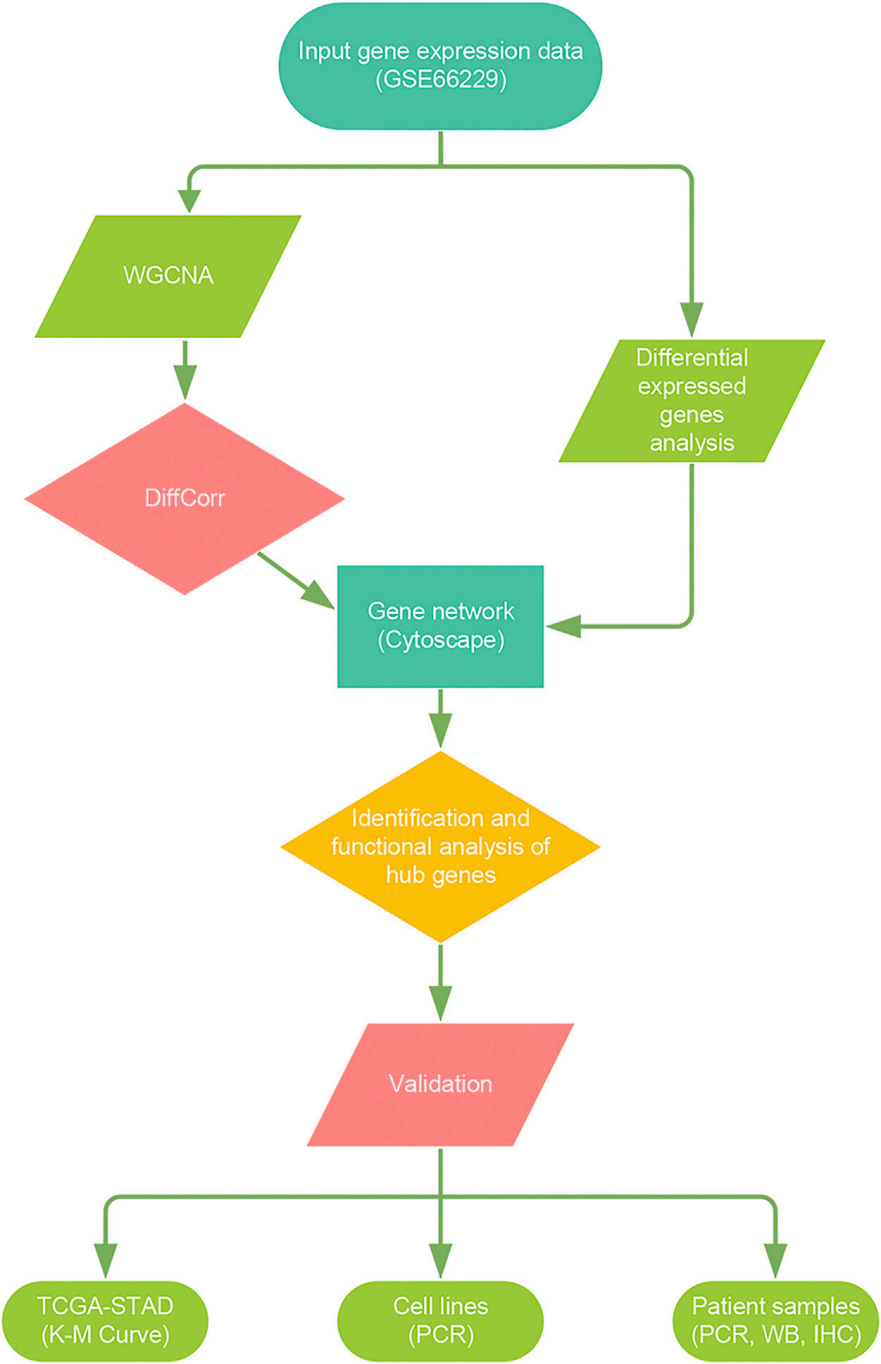
Flow diagram of the study. Data processing, analysis, and validation was shown in the picture.

The workflow of the data preparation, processing, analysis, and validation is shown in Figure 1. The raw data of microarray GSE66229 (tumor samples = 300, normal samples = 100) were downloaded from the Gene Expression Omnibus database (GEO: http://www.ncbi.nlm.nih.gov/geo/) and was further normalized by Robust multi-array average (RMA) using the R package “affy” (Gautier et al., 2004). The probes were concerted to gene symbols according to the platform GPL570 (Affymetrix Human Genome U133 Plus 2.0 Array). The Stomach adenocarcinoma (STAD) RNA-seq read counts data along with survival information was retrieved from The Cancer Genome Atlas database (TCGA, https://portal.gdc.cancer.gov/). After excluding samples without survival information, 387 samples were enrolled in this study. All analyses were carried out by R version 4.1.0.

### Construction of Weighted Gene Co-Expression Network

Gene expression profiles from GSE66229 were used to construct WGCNA using this package according to the protocol in R software (Langfelder and Horvath, 2008). First, we used the goodSamplesGenes (gsg) method to exclude samples with too many missing entries and genes with zero variance. Next, a similarity matrix between gene expression profiles was constructed based on pairwise Pearson correlation, which was converted to an adjacency matrix using a power adjacency function. The power was chosen based on the scale-free topology criterion according to the scale-free topology index (R2) as 0.9 (Zhang and Horvath, 2005). Afterwards, the adjacency matrix was transformed into a topological overlap matrix (TOM) to detect modules (Langfelder et al., 2008). Modules were cut using the Dynamic Tree-Cut algorithm (Langfelder et al., 2008). We cut genes into modules by (blockwiseModules) method with following parameters: minModuleSize = 30, mergeCutHeight = 0.25, deepSplit = 2, verbose = 3.

To extract co-expressed genes most related to GC carcinogenesis for further analysis, the modules and phenotypes were related by calculating the module eigengenes (MES), which were the representatives of all genes in a module. Modules with |ME|>0.5 were selected. In addition, we performed a correlation between MM (module membership) and GS (gene significance) in the selected modules, which reflected the overall relationships of all genes in the module with the phenotype. We focused on the modules which had a strong overall correlation between MM and GS (r > 0.5).

### Functional Enrichment of the Module Genes

Kyoto Encyclopedia of Genes and Genomes (KEGG) pathways analysis of the genes in the chosen module were performed using the R package “clusterProfiler” (Yu et al., 2012). Only the KEGG terms with FDR<0.05 were considered significant. After construction of the network, we used STRING database (https://cn.string-db.org/) to assist in researching relevant papers and functional analysis of the key genes and their co-expressed genes.

### Differential Correlation Analysis

DiffCorr is an R package to analyze and visualize differential correlations between two conditions in biological networks (Fukushima, 2013). Briefly, the analytical process of DiffCorr is divided in three steps. Firstly, different correlations were calculated via Fisher’s z-test. The Pearson correlations of a gene-pair under two conditions r_A_ and r_B_ were transformed respectively into Z_A_ and Z_B_ by formula $Z=\frac{1}{2}\log\frac{1+r}{1-r}$ . Differences were subsequently calculated following the formula $Z=\frac{Z_{A}-Z_{B}}{\sqrt{\frac{1}{n_{A}-3}+\frac{1}{n_{B}-3}}}$ , where n_A_ and n_B_ represented sample size under each condition. The local false-discovery rate (fdr) was used for controlling estimates. Secondly, eigen-molecule modules based on the first principal component (PC) were identified. Using these eigen-molecule modules, pair-wise differential correlations between genes can be tested. Finally, scaling and clustering was finished. Different pre-treatment methods, including auto-scaling (unit-variance scaling), range scaling, Pareto scaling, vast scaling, level scaling, and power transformation, were integrated with downstream differential correlation analyses.

### Construction and Visualization of the Gene Network

The pair-wise differential correlation results and DEA were integrated into Cytoscape software (version 3.7.1) to construct gene network. Using R package “limma” (Ritchie et al., 2015), DEA was used to decide whether genes were up-regulated or down-regulated in GSE66229.

Survival Analysis of the Key Genes in the Network.

The gene expression level was parsed into two groups and repeated for 91 times based on the cutoff value from 5 to 95 percent of its expression data. A repeated log-rank test based on each cutoff value was conducted, and a cutoff value with the lowest *p*-value was selected for Kaplan-Meier analysis using R package “survival” (HARRINGTON and FLEMING, 1982) and “survminer” (https://CRAN.R-project.org/package=survminer).

### Cell Lines and Cell Culture

The GC cell lines (BGC823, HGC27, MKN45, AGS, MGC803, SGC7901) and the human normal mucosal epithelium cell line GES-1 purchased from Chinese Academy of Sciences in Shanghai were cultured in a humidified atmosphere with 5% CO_2_ supplemented with 10% Fetal Bovine Serum (FBS), 100 IU/ml penicillin and 100 mg/ml streptomycin in a humidified atmosphere with 5% CO2 at 37°C.

### Patients and Tissues

Sixty-three pairs of GC tumor and corresponding adjacent normal tissues were collected from Zhongnan Hospital of Wuhan University. The patients had not experienced any chemotherapy or radiotherapy. All samples were obtained with informed patients’ consent before collection, and approved by the Zhongnan Hospital of Wuhan University Ethics Committee. Samples were snap-frozen and stored at−80°C until use in real-time qPCR (RT-qPCR) and WB (western blot) experiments. In addition, we conducted immunohistochemical (IHC) staining of formalin-fixed paraffin-embedded GC patients and normal control samples.

### RNA Isolation and Quantitative Real-Time PCR

Total RNA was extracted from GC cell lines and tissues using the Trizol reagent (Invitrogen, United States) according to the manufacturer’s protocol. RNA concentration was measured by NanoDrop ultramicroscopy spectrophotometer 2000 (Thermo Fisher Scientific, United States). 1 ug RNA was reverse transcribed to cDNA using Primescript™ RT reagent kit (Vazyme, China). Quantitation of mRNA expression levels were performed on a Bio-Rad IQ5 Real-Time PCR instrument (Bio-Rad, United States) using SYBR-Green PCR Master Mix (Vazyme, China). The primer sequences are listed in Supplementary Table S1.

### Western Blot Analysis

Cells were lysed using RIPA buffer containing protease inhibitor cocktail (Thermo Fisher Scientific, United States). The proteins were separated utilizing 10% sodium dodecyl sulfate-polyacrylamide gels and then transferred to a polyvinylidene fluoride membrane (Millipore, United States). After 2 hours of protein blocking with 5% non-fat milk, the membranes were incubated with primary antibodies and HRP-conjugated secondary antibodies. Proteins were detected using Bio-Rad Image Lab software. Quantitation on western blots have been performed using ImageJ software. The following primary antibodies were used: anti-GEMIN5 (1:1,000, Abcam ab201691), anti-PDFN2 (1:1,000, Abcam ab237534), anti-β-actin (1:5,000, Abcam ab8226).

### Immunohistochemistry

Formalin-fixed tissues were paraffin embedded and sectioned (5-μm-thick sections). Antigen retrieval was performed by microwave oven for 18 min in citrate buffe (Beyotime, China). 3% hydrogen peroxide (Merck, Germany) was used to block endogenous peroxidase activity. Nonspecific staining was blocked followed by incubation with antibodies to GEMIN5 (1:100, Abcam ab201691) and PFDN2 (1:100, Abcam ab237534). Immunostaining was performed using DAB according to the manufacturer’s instructions.

## Results

### Construction of Weighted Gene Co-Expression Network

**FIGURE 2:**
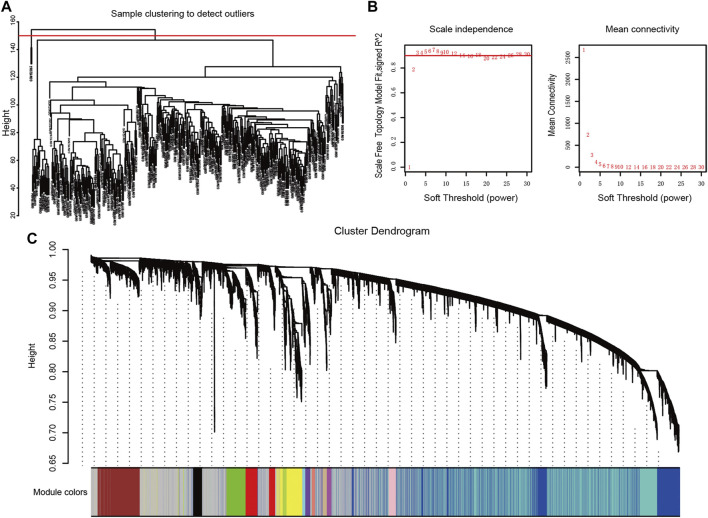
Construction of weighted gene co-expression network **(A)** Cluster dendrogram displays the relationship between samples **(B)** The scale-free fit index for soft-thresholding powers. Left: the relationship between the soft-threshold and scale-free R2. Right: the relationship between the soft-threshold and mean connectivity. Different modules are labeled in different colors **(C)** Dendrogram of all genes clustered in GSE66229.

After excluding two abnormal samples, GSM1523817 and GSM1523984 (Figure 2A), we performed WGCNA analysis to detect clusters of genes most correlated with GC carcinogenesis based on the expression profiles of the remaining 398 samples in GSE66229. The power, a critical parameter in the analysis, was chosen to be three to ensure a scale-free network (*R* ^2^ = 0.9, Figure 2B). A dendrogram of all genes (n = 16,241) was clustered using the average linkage method and Biweight midcorrelation (Bicor) method (Figure 2C). A total of eighteen modules with widely varied numbers of co-expressed genes were identified through hierarchical clustering. The genes and their attributed modules are listed in Supplementary Table S1.

### Identification of Significant Modules in WGCNA

**FIGURE 3:**
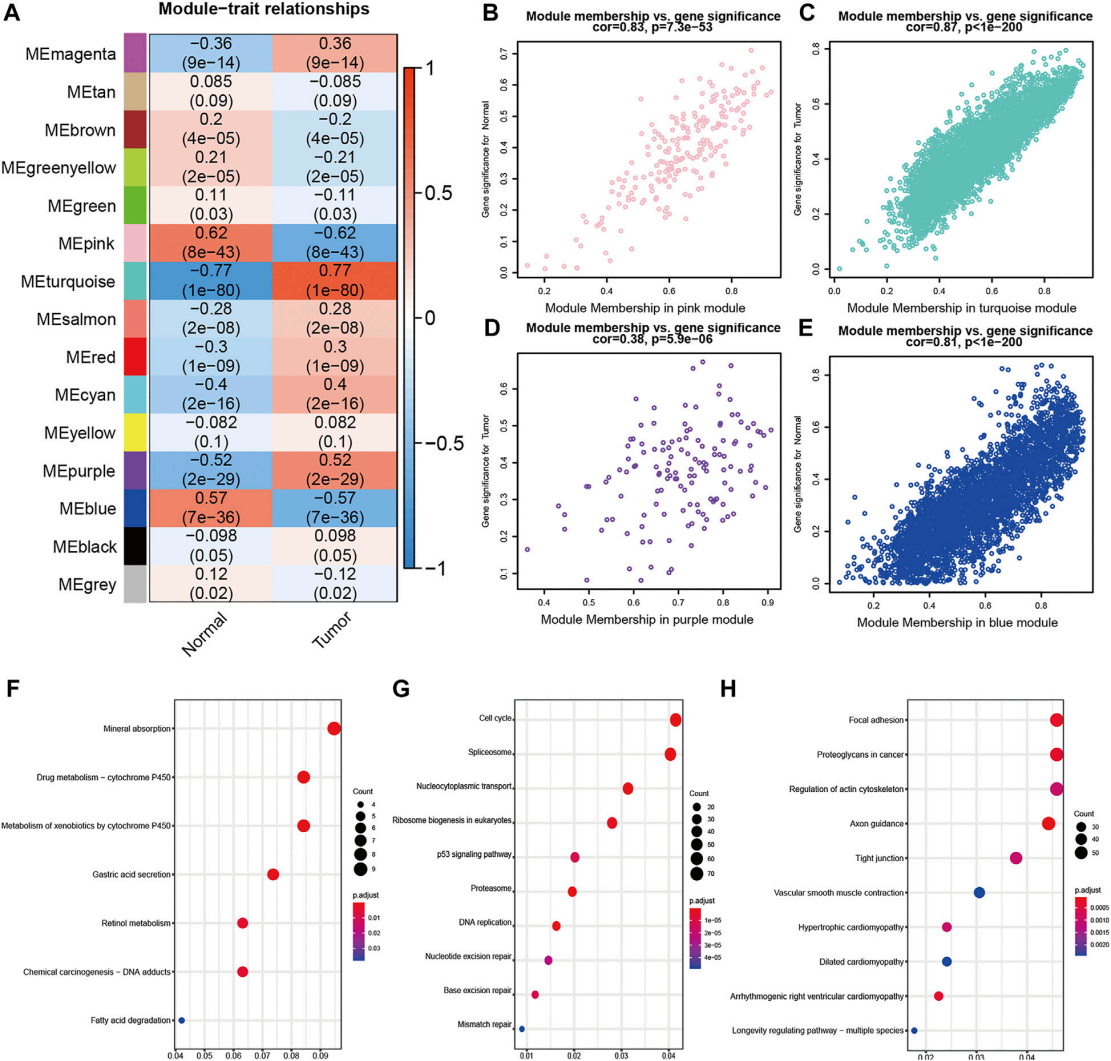
Identification of significant modules in WGCNA **(A)** Heatmap of the correlation between module eigengenes and disease status. Each cell contained the correlation coefficients and *p* value. Red indicates positive correlations while blue indicates negative **(B)–(E)** Scatterplots of Gene Significance (GS) for disease vs Module Membership (MM) in the pink **(B)**, turquoise **(C)**, purple **(D)** and blue **(E)** modules. There was a highly significant correlation between GS and MM in pink, purple and blue module **(F)–(H)** KEGG pathway analysis in pink **(F)**, turquoise **(G)** and blue **(H)** modules.

The relationship between modules and GC was subsequently explored (Figure 3A). Four modules presented a strong relationship with correlation coefficients above 0.5, which were the pink module (r = −0.62, p = 8e−43), the turquoise module (r = 0.77, p = 1e−80), the purple module (r = 0.52, p = 2e−29) and the blue module (r = −0.57, p = 7e−36).

Next, to further screen modules with most genes associated with GC, we performed a correlation analysis between GS and MM (Figures 3B–E). The pink module (r = 0.83, *p* = 7.3e−53), the turquoise module (r = 0.87, p < 1e−200) and the blue module (r = 0.81, p < 1e−200) were chosen for further analysis, while the purple module (r = 0.38, *p* = 5.9e−06) was excluded.

Functional analysis of genes in the three modules was performed by KEGG pathway analysis (Supplementary Table S2). The top five significant pathways in the pink module were mineral absorption, drug metabolism − cytochrome P450, metabolism of xenobiotics by cytochrome P450, gastric acid secretion and retinol metabolism (Figure 3F). The top five significant pathways in the turquoise module were cell cycle, spliceosome, nucleocytoplasmic transport, ribosome biogenesis in eukaryotes and p53 signaling pathway (Figure 3G). The top five significant pathways in the blue module were focal adhesion, proteoglycans in cancer, regulation of actin cytoskeleton, axon guidance and tight junction (Figure 3H).

### Calculation of Differential Correlations and Visualization of the Gene Network

**FIGURE 4:**
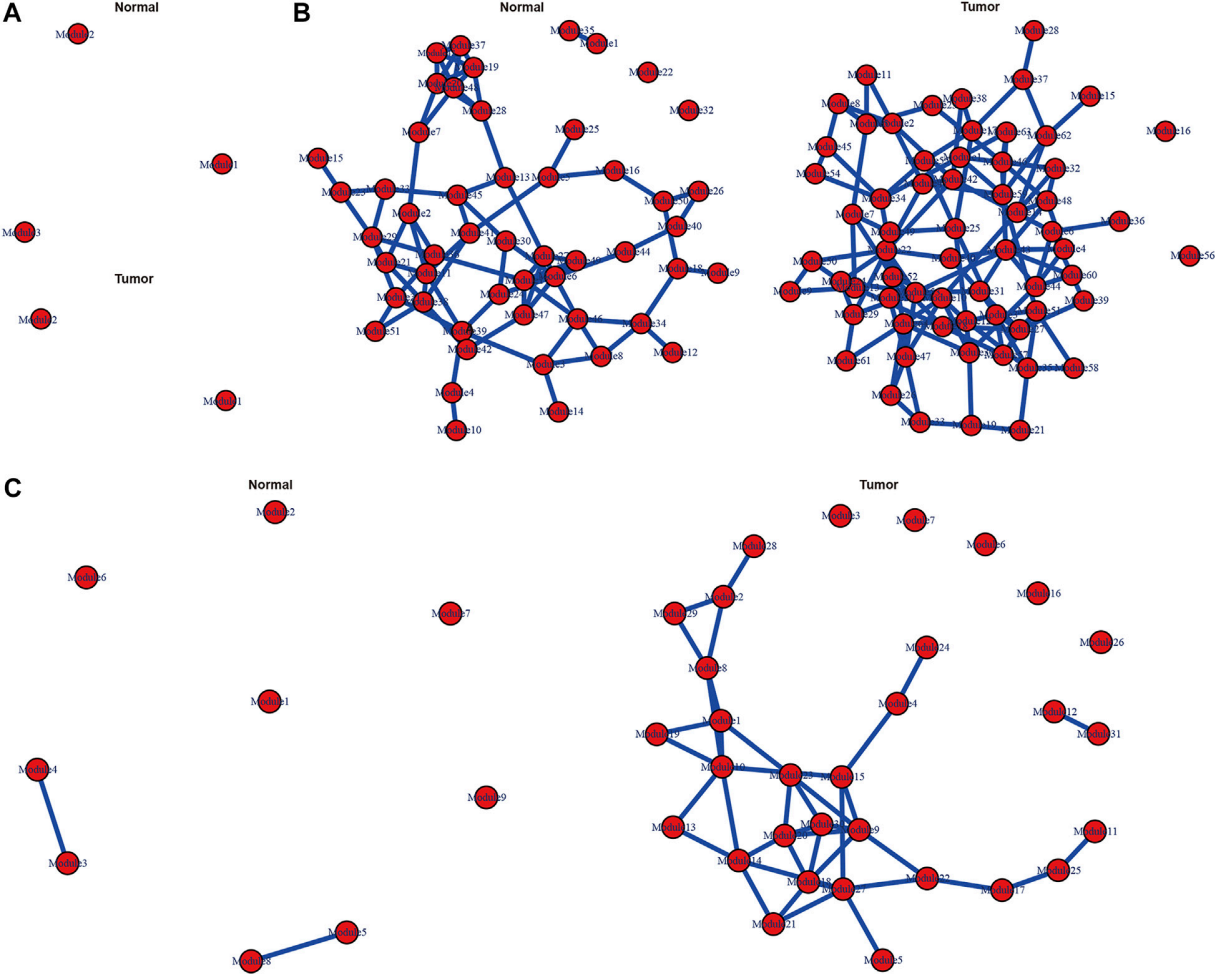
Representations of the module network and differential co-expressions. Images of pink **(A)**, turquoise **(B)** and blue **(C)** module networks including cancerous and normal samples.

Genes in pink, turquoise and blue modules were chosen to analyze their differential correlations separately. First, we used (cluster.molecule) function to cluster genes based on their expression profiles. One-correlation coefficient was adopted to measure distance (the cutoff of the coefficient was 0.6) according to the (cutree) function. Then (get.eigen.molecule) and (get.eigen.molecule.graph) were applied to visualize this process (Figure 4). Finally, the results of pair-wise differential correlations were exported via (comp.2. cc.fdr) function (Supplementary Table S3), and the top 10 significantly differential coexpressions (FDR <0.05) in each module were shown in Table 1.

**TABLE 1:** Top 10 correlated gene pairs changed to the opposite direction in each module between normal and GC samples.

molecule_X	molecule_Y	r1	r2	Lfdr	Module color
MARC-2	AKIP1	−0.6314071	0.7333939	1.96E-15	pink
MT1G	AKIP1	−0.6576778	0.7285792	1.96E-15	pink
XYLT2	AKIP1	−0.4606301	0.7007189	1.96E-15	pink
AKR1C1	OGFOD1	−0.6987339	0.6827796	1.96E-15	pink
MT1E	AKIP1	−0.6681788	0.6715482	1.96E-15	pink
KCNJ16	OGFOD1	−0.7569397	0.6696279	1.96E-15	pink
USP31	CLIC6	−0.5173597	0.6574277	1.96E-15	pink
NEDD4L	DNM1L	−0.6721752	0.6508082	1.96E-15	pink
SLC7A8	DNM1L	−0.659538	0.6540433	1.96E-15	pink
SMIM14	AKIP1	−0.4792319	0.6367244	1.96E-15	pink
GEMIN4	TSR1	−0.4024842	0.8245598	2.83E-13	blue
AAR2	STAU1	−0.4362916	0.7703166	2.83E-13	blue
PFDN2	NUF2	−0.439736	0.7305703	2.83E-13	blue
PFDN2	KIF14	−0.4129973	0.7173457	2.83E-13	blue
PUF60	ATAD2	−0.4779598	0.7099839	2.83E-13	blue
LIG1	PRPF31	−0.4229943	0.7096661	2.83E-13	blue
RECQL4	C8orf33	−0.4263906	0.7094297	2.83E-13	blue
AAR2	RALGAPB	−0.4108851	0.6992478	2.83E-13	blue
ILKAP	DTYMK	−0.4058326	0.6831076	2.83E-13	blue
NXT1	CSNK2A1	−0.4242544	0.6767982	2.83E-13	blue
SLC4A2	GPR155	0.7119232	−0.4382693	1.54E-14	turquoise
NARS	TXNL1	−0.6924692	0.433835	1.54E-14	turquoise
MAN1A1	GPR155	−0.6713351	0.4305737	1.54E-14	turquoise
NARS	DYM	−0.6363036	0.4233434	1.54E-14	turquoise
ZNF385B	ABCB1	−0.6213381	0.4234794	1.54E-14	turquoise
MPP1	GPR155	−0.680156	0.4836556	1.54E-14	turquoise
WWC1	GPR155	0.5676928	−0.4088423	1.54E-14	turquoise
IPPK	GPR155	0.5706811	−0.4198478	1.54E-14	turquoise
SCG5	GPR155	−0.570117	0.449409	1.54E-14	turquoise
TYRP1	FZD4	−0.5218242	0.413089	1.54E-14	turquoise

**FIGURE 5:**
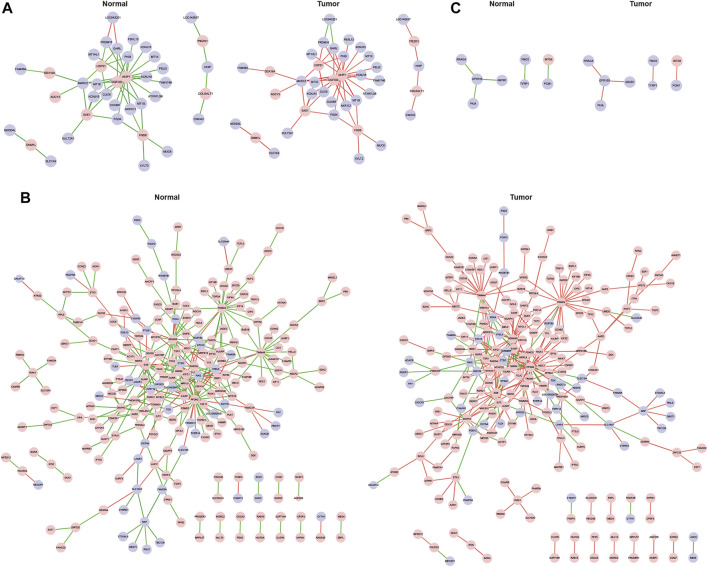
Differentially co-expressed gene networks in the pink **(A)**, turquoise **(B)** and blue **(C)** modules. Each node represented a gene, with lavender-filled color denoting decreased genes and pink-filled color denoting increased genes in GC. The edge represented connection between two genes. The green edge represented negative correlation and red positive correlation.

The DiffCorr package also detected oppositely correlated gene pairs where two molecules exhibit positive correlation in one condition and negative correlation in the other condition, which referred to as a “switching mechanism” (Kayano et al., 2011). These switched gene pairs were of concern in the pathogenesis of GC. As the correlation relationships of gene pairs in each module were complicated, we select gene pairs which had both at least moderately strong positive and negative correlations (correlation r > 0.5) to construct a gene network. In total, we obtained 52 oppositely correlated gene pairs from pink module, 307 gene pairs from turquoise module and five gene pairs from blue module (Supplementary Table S4), and the gene network based on which was presented in Figure 5 by cytoscape software.

### Functional Analysis of Key Genes w ith Differential Correlations

In the pink module, AKIP1 deserved more attention than other genes (Figure 5A). In this module, AKIP1 appeared in most gene pairs and linked to 19 other genes. AKIP1 was increased in GC and its co-expressed genes were mostly decreased in GC. Previously, AKIP1 has been reported to act as an oncogene in GC by activating Slug-induced EMT and AKIP1 significantly correlated with clinical metastasis and poor prognosis (Chen et al., 2019). By uploading AKIP1 and its co-expressed genes into string database, we found that its co-expressed genes KCNJ15, KCNJ16, GHRL and CCKBR were all involved in gastric acid secretion (Rau et al., 2013). Thus, our research enhanced the understanding of the role of AKIP1 in GC.

In the turquoise module, GEMIN5, PFDN2 and Sjogren syndrome antigen B (SSB) were in the center part of the network and possessed more edges than other genes (Figure 5B). These three genes were all increased in GC. Gem Nuclear Organelle Associated Protein 5 (GEMIN5), a component of the spliceosomal complex, plays a crucial role in mRNA splicing and can affect tumor cell motility (Lee et al., 2008). However, it has not yet been studied in GC. Its co-expressed genes SHMT2 and MTHFD2 are two mitochondrial enzymes which take part in folate metabolism and play critical roles in the gastrointestinal cancer survival and proliferation (Konno et al., 2017), which indicated that GEMIN5 might function as an oncogene in GC by exerting impacts on folate embolism. Hence, our study discovered for the first time, the critical role of GEMIN5 in GC and provided information for further function research.

As an autoimmune RNA-binding protein, SSB binds to the 3′ poly(U) terminus of nascent RNA polymerase III transcripts, protecting them from exonuclease digestion and facilitating their folding and maturation (Chambers et al., 1988). It has been recently identified as a pre-miRNA-binding protein that regulates miRNA processing *in vitro*, and was correlated with dicer in human cancer transcriptome and prognosis (Liang et al., 2013).

However, the role of SSB was still unclear in human cancers. Thus, our network provided information for further investigation.

PFDN2 belongs to the prefoldin subunits family which can bind and stabilize newly synthesized polypeptides (Mo et al., 2020). In GC, high mRNA expression of PFDN2 displayed poor OS(Yesseyeva et al., 2020). Its co-expressed genes TTK, CCNB2, PLK1 were in the cell cycle pathway, indicating PFDN2 might play an important role in GC via affecting cell cycle. Therefore, our research firstly validated the differential expression of PFDN2, and identified its co-expression genes in GC which provided valuable information for further functional mechanism explore.

In the blue module, GPR155 (G protein-coupled receptor 155) stood out in the network (Figure 5C). In GC, GPR155 transcription was suppressed in GC cell lines compared with a nontumorigenic cell line, and low GPR155 mRNA level was an independent biomarker of hematogenous metastasis (Shimizu et al., 2017). Consistent in our study, the expression of GPR155 was downregulated in gastric cancer tissue of the ACRG cohort.

### Validation of Novel Key Genes of GC

**FIGURE 6:**
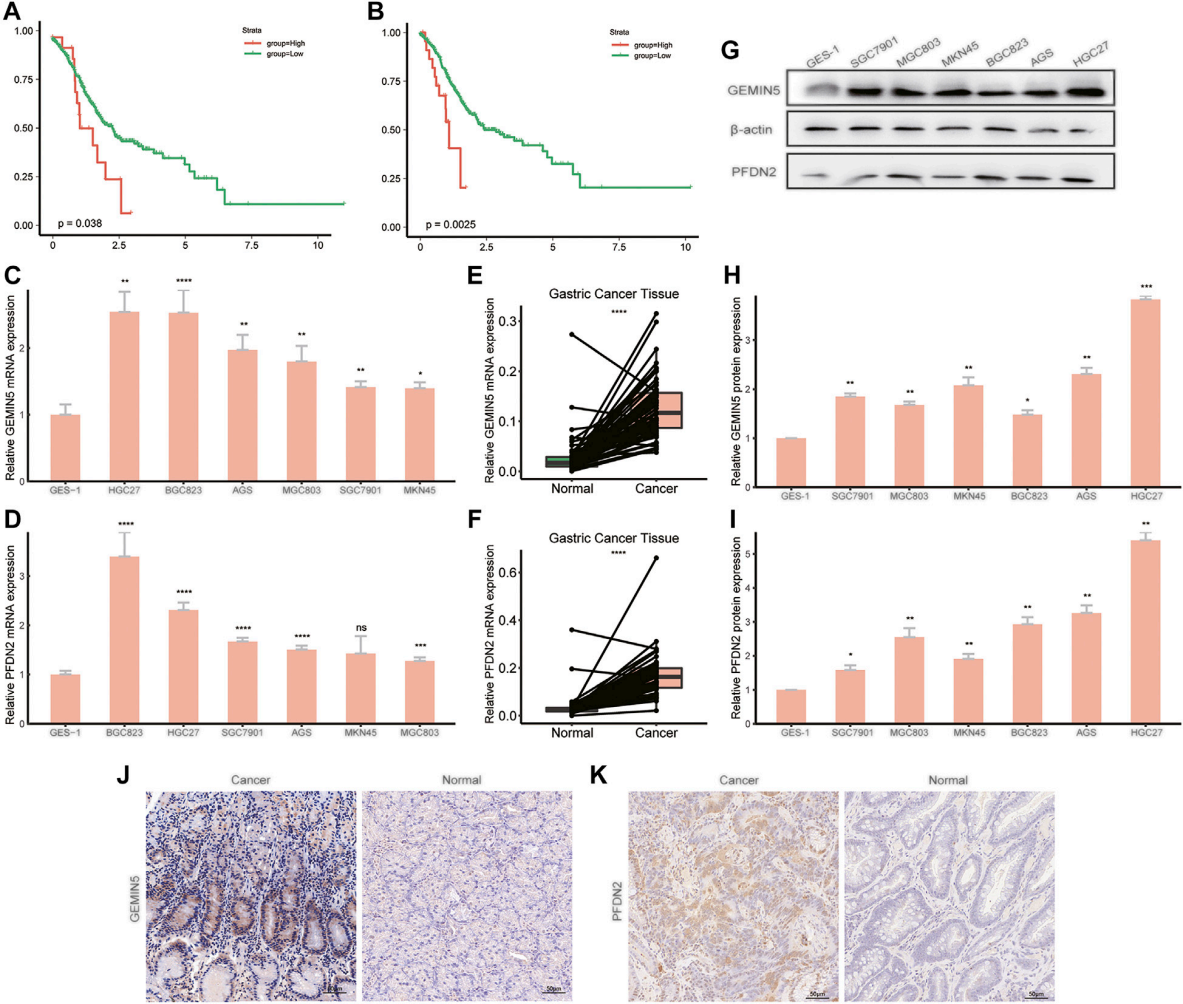
Validation of the key genes using TCGA-STAD data and laboratory experiments. Kaplan-Meier curve analysis of GEMIN5 **(A)** and PFDN2 **(B)** was shown. Expression of GEMIN5 and PFDN2 mRNA levels were validated by RT-qPCR in cell lines **(C)–(D)** and patient samples **(E)–(F)**. Expression of GEMIN5 and PFDN2 protein levels were validated by western blot **(G–I)** and immunohistochemistry **(J–K)**. Both mRNA and protein expression level of GEMIN5 and PFDN2 were higher in tumor samples and cancer cell lines than in normal samples and nontumorigenic cell line.

In order to validate the two novel possible key genes, GEMIN5 and PFDN2, we performed Kaplan-Meier analysis in TCGA-STAD dataset. RT-qPCR, WB, and IHC experiments were used to detect mRNA or protein expression in GC cell lines and patient samples. The Kaplan-Meier analysis showed higher levels of GEMIN5 and PFDN2 correlated with poor prognosis in GC patients (Figures 6A,B). RT-qPCR results showed that the mRNA expression level of GEMIN5 and PFDN2 were higher in cancer cells of HGC27, BGC823, AGS, MGC803, SGC7901, and MKN45 than in human normal mucosal epithelium cells GES-1 (Figures 6C, D).Also, the mRNA expression level of GEMIN5 and PFDN2 were higher in GC tissues than in normal gastric tissues (Figures 6E, F). The protein expression level of GEMIN5 and PFDN2 were confirmed consistent with mRNA expression levels in the same cell lines and patient tissues by WB (Figures 6G–I) and IHC (Figures 6J, K). In addition, we performed correlation analyses between the mRNA expression level and clinicopathological characteristics in our own patient samples (Table 2). High GEMIN5 expression was significantly correlated to tumor size (*p* = 0.031), T-stage (*p* = 0.009), lymphatic metastasis (*p* = 0.001), TNM stage (*p* = 0.002) and histological grade (*p* = 0.034). High PFDN2 expression was significantly correlated to age (*p* = 0.027), T-stage (*p* = 0.047), TNM stage (*p* = 0.05). For the first time, our experiments validated the elevated expression of GEMIN5 and PFDN2 in GC cell lines and GC samples and assumed their critical roles in the pathogenesis of GC.

**TABLE 2:** clinicopathological features and expression of PFDN2 and GEMIN5

Characteristics	PFDN2 expression		*p* Value	GEMIN5 expression		*p* Value
	Low	High		Low	High	
Total cases	31	32	—	31	32	—
Gender						
Male	22	23	—	22	23	—
Female	9	9	0.936	9	9	0.936
Age(years)						
<60	12	10	—	15	7	—
≥60	19	22	0.535	16	25	0.027
Tumor size (cm)						
<5	26	19	—	22	23	—
≥5	5	13	0.031	9	9	0.936
T-stage						
T1/T2	10	2	—	9	3	—
T3/T4	21	30	0.009	22	29	0.047
Distant Metastasis						
Yes	3	5	—	2	6	—
No	28	27	0.478	29	26	0.143
Lymphatic Metastasis						
N0/N1	22	8	—	16	14	—
N2/N3	9	24	0.001	15	18	0.532
TNM stage						
I/II	16	5	—	14	7	—
III/Ⅳ	15	27	0.002	17	25	0.05
Histological grade						
Well/Moderate Dif	8	2	—	7	3	—
Poorly Dif	23	30	0.034	24	29	0.152

## Discussion

Previous studies have revealed gene interaction networks in multiple cancers based on their co-expression patterns (Liu et al., 2020; Nangraj et al., 2020; Bo et al., 2021), but little is known about differential correlations between disease status and health status, which could be an instructive view to elucidate deep mechanisms of pathogenesis of cancer. It is a growing direction in co-expression analysis that researchers have begun to develop tools focused on genes that are correlated under one condition but show little or no correlation in another condition, including coXpress (Watson, 2006), DCGL (Yang et al., 2013), and DiffCorr (Fukushima, 2013; Yang et al., 2013). CoXpress is a simple method to identify differentially co-expressed gene groups and should be used as first step in the analysis of co-expression (Watson, 2006). DCGL selects differentially regulated genes (DRGs) and differentially regulated links (DRLs) according to the transcription factor (TF)-to-target information (Yang et al., 2013). DiffCorr identifies pattern changes between two experimental conditions in correlation networks and can be used to detect biomarker candidates (Fukushima, 2013).

However, there is plenty of scope for this approach in cancer research. In this study, for the first time, we constructed an *in silico* gene network in GC benefiting from the availability of large-scale data and multiple algorithms to calculate differential correlations between gene pairs. First , we used WGCNA as a gene filtration to detect significant co-expressed genes modules and narrowed down the range of suspects. Secondly, we used DiffCorr to identify differentially co-expressed gene modules inside significant modules. To identify the key genes in GC, we analyzed the network focusing on biological functions. A recent study in plant biology has used DiffCorr to identify a novel community of genes to explain differences in leaf phenotypes which has no enriched GO terms (Nakayama et al., 2021). We carried out our functional research of our key genes by studying the co-expressed genes and referring to previous researches with the help of STRING database. Candidate key genes were validated using clinical information and laboratory experiments. In addition, both candidate key genes and their differentially co-expressed genes could be useful guidance for further experimental investigations and eventually facilitating diagnosis and treatment on GC patients.

The most significant gene in the pink module A-kinase-interacting protein 1 (AKIP1) is a nuclear protein known to interact with the catalytic subunit of PKA (PKAc) and a binding partner of NF-kappaB p65 subunit (Gao et al., 2008). Due to its function, AKIP1 has previously been reported to act as a potential oncogenic protein in various cancers such as esophageal squamous cell carcinoma (Lin et al., 2015), breast carcinoma (Mo et al., 2016), non-small-cell lung cancer (Guo et al., 2017) and colorectal cancer (Jiang et al., 2018). Our research reconfirmed that it played an important role in GC and enhanced understanding by revealing its co-expressed genes.

The key gene GPR155 in the blue module has also been extensively studied in cancers. In addition to its role as a biomarker in GC, the downregulation of GPR155 was reported as significantly associated with more aggressive HCC phenotypes including high preoperative α-fetoprotein, poor differentiation, serosal infiltration, vascular invasion, and advanced disease stage (Umeda et al., 2017). The presence of GPR155 in multiple gene pairs suggest it might be a crucial regulatory gene in GC and this offers clues for carrying out its further research in cancers.

With regards to the turquoise module, we identified two novel key genes GEMIN5 and PFDN2. It has been reported that GEMIN5 was the most differentially varied protein in breast cancer cell lines after modulation of Nm23-H1, which is the first MSG to be characterized (Lee et al., 2009). A recent study has found that gain of PFDN2 somatic copy-number was associated with poor survival in patients with metastatic urothelial carcinoma treated with platinum-based chemotherapy (Riester et al., 2014). However, the significance of GEMIN5 and PFDN2 was hardly mentioned in GC. Our network offered clues that they might possess significant position in the pathogenesis of GC, because they linked to most genes in the co-expressed genes module and might serve as key regulators. In addition, we validated their differential expression in mRNA and protein level in laboratory and analyzed their correlations with clinicopathological features.

Conventional methods to identify key genes between disease and health status require complex screening from a list of hundred candidate genes, whereas in our research, we narrowed down the range of suspects by WGCNA and DiffCorr and achieved effective results. Our research is perspective and efficient in finding novel biomarkers in cancers. However, only transcriptomics data was enrolled in our analytical process, which covered only one single layer of genome. In future studies, further researches should be carried out on differential correlations integrating multidimensional data such as proteomic data and single-cell sequencing data.

## Data Availability

The datasets presented in this study can be found in online repositories. The names of the repository/repositories and accession number(s) can be found in the article/Supplementary Material.
