# Supplementary material for: Identification and Validation of Key Genes of Differential Correlations in Gastric Cancer
Source: Front Cell Dev Biol. 2022 Jan 13;9:801687. doi: 10.3389/fcell.2021.801687 (PMC8794754; doi:10.3389/fcell.2021.801687)
Supplement: Supplementary file 3 [file Table1.DOCX]

Table S1 The sequences of primers for qRT-PCR

| Gene | Primer Sequence (5’ – 3’) |
| --- | --- |
| GEMIN5 | F: CAAACAGCTCCTGCTTCACA  R: TAGGTGGTCACAGCCATCAG |
| PFDN2 | F: ATGAGCACAGCCTAGTGATCG  R: ACTCCTCCAACCATGCGGTA |
| GAPDH | F: TCACCACCATGGAGAAGGC  R: GCTAAGCAGTTGGTGGTGCA |
