# Supplementary figures and images for: Identification and Validation of Key Genes of Differential Correlations in Gastric Cancer
Source: Front Cell Dev Biol. 2022 Jan 13;9:801687. doi: 10.3389/fcell.2021.801687 (PMC8794754; doi:10.3389/fcell.2021.801687)

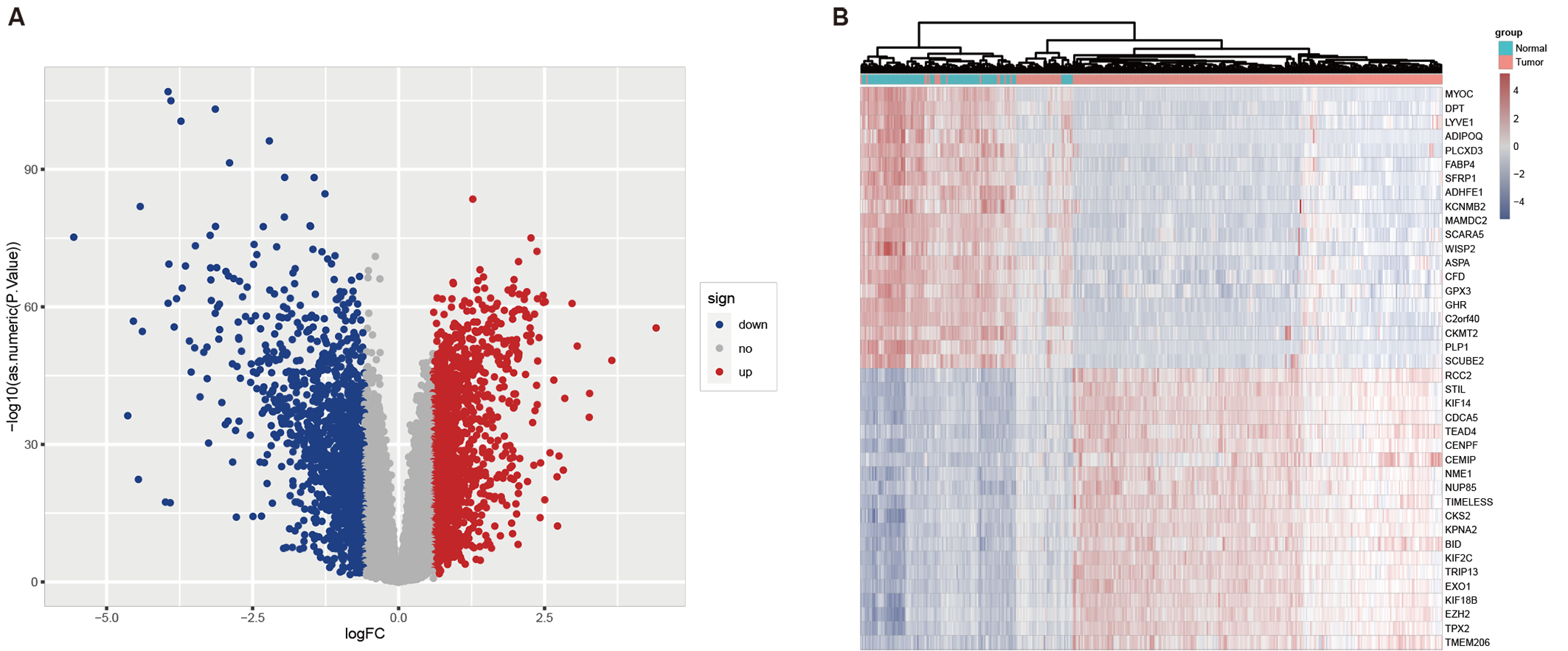

Supplement: Supplementary file 4 [file Image1.TIF]
